# How much do you know about benign, preneoplastic, non-invasive and invasive neoplastic lesions of the urinary bladder classified according to the 2004 WHO scheme?

**DOI:** 10.1186/1746-1596-6-31

**Published:** 2011-04-07

**Authors:** Rodolfo Montironi, Liang Cheng, Marina Scarpelli, Roberta Mazzucchelli, Antonio Lopez-Beltran

**Affiliations:** 1 Section of Pathological Anatomy, School of Medicine, Polytechnic University of the Marche Region (Ancona), Ancona, Italy; 2 Department of Pathology and Laboratory Medicine, Indiana University School of Medicine, Indianapolis, IN, USA; 3 Unit of Anatomic Pathology, Cordoba University Medical School, Cordoba, Spain

## Abstract

The aim of this essay is the self assessment of the level of knowledge of the 2004 WHO classification of bladder neoplasms through a series of MCQs, each associated a short commentary. This paper is directed to all who are involved with the application of this classification at the anticancer research, diagnostic, prognostic and therapeutic levels, in particular to uropathologists, urologists and oncologists.

## Introduction

The 1973 WHO histological grading of bladder cancer is one of most successful grading systems among all organ sites and has been validated since its introduction three decades ago. In 1998, a system of classifying non-invasive flat and papillary urothelial neoplasms of the urinary bladder was proposed by the *International Society of Urologic Pathology *in association with the *World Health Organization*. This became known as the 1998 WHO/ISUP classification system [1]. In 2004, this classification system was adopted in *Pathology and Genetics of Tumours of the Urinary System and Male Genital Organs*, one of a series of WHO "Blue Books" for the classification of tumours (Additional file 1: Appendix 1). This is known as the ***2004 WHO classification ***[2].

This paper is directed to all who are involved with the application of this classification at the anticancer research, diagnostic, prognostic and therapeutic levels, in particular to uropathologists, urologists and oncologists. The aim of this essay is to test the level of knowledge of the 2004 classification through a series of MCQs, each associated a short commentary. Additional file 2: Appendix 2 can be used by the readers to record the correct answers. The list with the correct answers will be published in the next journal issue. Those interested to discuss the MCQs with the authors of this contribution should contact the corresponding author.

## Multiple choice questions and explanatory notes

### Question No 1. *Normal urothelium. Which of the followings items is wrong?*

1. It is the type of epithelium lining the urinary bladder, ureters, and renal pelvis

2. Its thickness varies with the state of distension of the bladder

3. By immunohistochemistry it shows reactivity for CK20 only in the basal cell layer

4. Frequently technical problems such as tangential cut, thick sections and vagaries of staining and fixation may cause the normal urothelium appear hyperchromatic and hyperplastic

#### Explanatory notes

**Figure 1:**
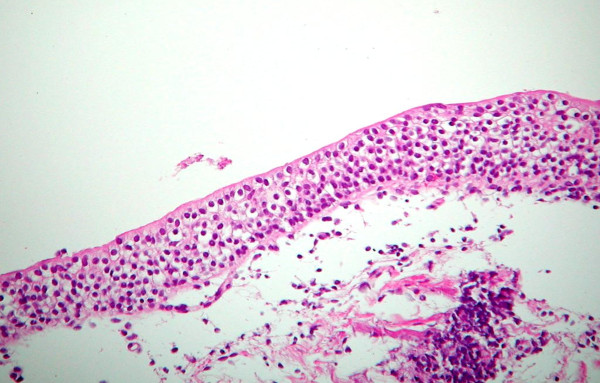
**Normal urothelium**.

Urothelium (Figure 1) is a multilayered epithelium of the urinary bladder, ureters, and renal pelvis in which the cells mature to form the very large surface "umbrella cells." The thickness of the normal urothelium varies with the state of distension of the bladder (2 to 4 cell layers when dilated and 5 to 7 layers when contracted) [3]. The urothelium of the renal pelvis, urethra and the bladder neck is usually composed of slightly larger cells, which have diminished cytoplasmic clearing and hence may be misinterpreted as dysplasia. Umbrella cells may show some degree of nuclear pleomorphism, which should not be misconstrued to be dysplastic. If the sections are thick, the urothelium may appear hyperchromatic and this artifact compounded with tangential sectioning may result in changes felt to represent dysplasia. Vagaries of staining and fixation may also impart hyperchromasia to benign nuclei. Normal urothelium shows reactivity for CK20 only in the superficial umbrella cell layer, while CD44 staining is limited to the basal and parabasal urothelial cells. Nuclear staining for p53 is absent in normal urothelium [4].

### Question No 2. *Flat urothelial hyperplasia. Which of the following items is correct?*

1. The diagnosis does not usually requires counting the number of cell layers in the urothelium

2. It consists of a markedly thinned urothelium, lower than seven cells layers thick, with minimal cytological atypia

3. When seen by itself, there are data proving that it has premalignant potential

4. It is a reactive process unrelated to bladder cancer

#### Explanatory notes

**Figure 2:**
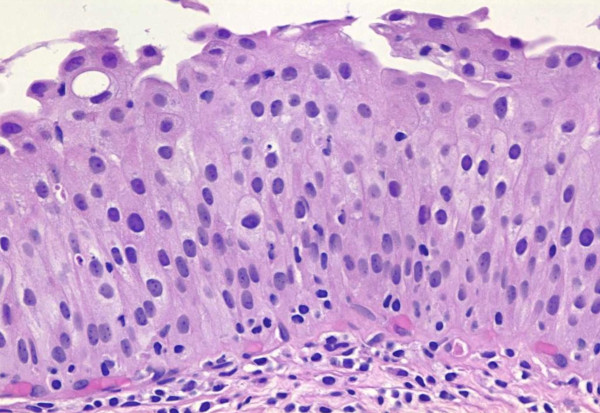
**Flat urothelial hyperplasia**.

Historically the term "hyperplasia" has been equated with counting cell layers and specifically considering the epithelium to be hyperplastic if there were more than 7 cell layers (Figure 2). It is well recognized that the apparent number of cell layers in the normal urothelium is variable and dependent on the state of contraction of the bladder wall. The 2004 classification recognizes hyperplasia as when there is a "markedly thickened mucosa without atypia." Counting cell layers is not recommended. The relationship between hyperplasia and neoplasia is unknown [2,5,6].

### Question No 3. *Reactive atypia. Which of the followings is correct?*

1. It is a preneoplastic lesion

2. It is a synonymous of flat dysplasia

3. It consists of cytological abnormalities occurring in acutely or chronically inflamed urothelium

4. It never coexists with dysplasia or in situ carcinoma

#### Explanatory notes

**Figure 3:**
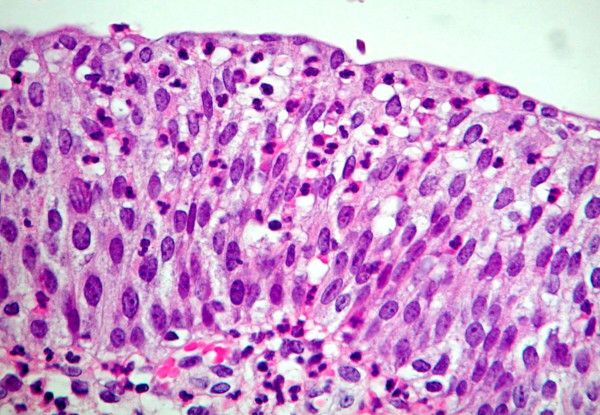
**Reactive atypia**.

In the presence of acute and/or chronic inflammation, the urothelium shows a wide range of reactive changes (Figure 3). There is usually a history of instrumentation, infection or treatment with intravesical agents. Some patterns of atypia are associated with specific aetiologies. In reactive atypia the epithelium may or may not be thickened. Although a thickened epithelium is typically associated with a reactive process, carcinoma in situ can also produce a thicker than normal epithelium. Nuclei are uniformly enlarged, vesicular, and may have prominent usually centrally located nucleoli. Mitoses may be frequent and are in the lower epithelial layers. Inflammation is almost always present [2].

### Question No 4. *Papillary urothelial hyperplasia (Pseudopapillary hyperplasia). Which of the followings is correct?*

1. Well-developed branching fibrovascular cores are present

2. Urothelium exhibiting a slight "tenting", undulating, or papillary growth; often there are one or several dilated capillaries at the base of the lesion

3. It is not a preneoplastic lesion/condition

4. This term is also used to describe polypoid cystitis

#### Explanatory notes

**Figure 4:**
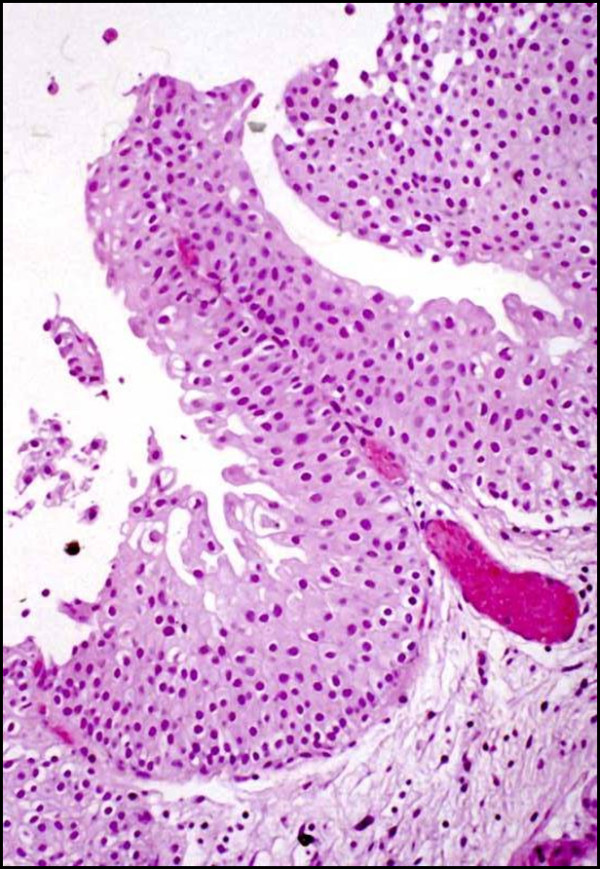
**Papillary urothelial hyperplasia (pseudopapillary hyperplasia)**.

In the 1998 WHO/ISUP classification, papillary hyperplasia was included as a category with the group of papillary lesions [1]. In the 2004 WHO classification, this is no longer included as a specific designation but it is recognized that hyperplasias may be flat or pseudopapillary (Figure 4) [2]. In the current classification, hyperplasia with a pseudopapillary architecture refers to a slight tenting or undulation of the urothelium lacking a well defined central fibrovascular core, although small vessels may be present at the base of the papillae. There is no significant cytologic or architectural atypia. These have most often been described in the setting of known papillary neoplasia. When identified de novo the significance regarding subsequent development of neoplasia is unknown.

### Question No 5. *Urothelial dysplasia Which of the followings is correct?*

1. There is no evidence that dysplasia may be a precursor of invasive carcinoma

2. The natural history of dysplasia in humans is poorly understood. There is some evidence that dysplasia may be a precursor of invasive carcinoma

3. It consists of a markedly thickened urothelium, greater than seven cells layers thick, with cytological atypia

4. The thickness of the urothelium and the polarity of the cells are maintained

#### Explanatory notes

**Figure 5:**
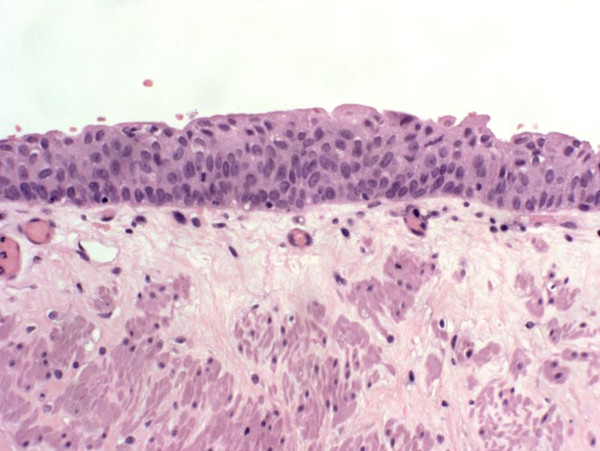
**Urothelial dysplasia**.

Histologically there is some architectural distortion. The nuclei are irregularly enlarged with some hyperchromasia and pleomorphism present. Overall the features are those of a neoplastic atypia but fall short of the criteria for carcinoma in situ outlined below (Figure 5). This category also suffers from a significant problem in diagnostic reproducibility. The natural history of lesions with dysplastic features of a lesser degree than the moderate to severe categories is unknown. There is however some evidence, largely genetic that it shares some abnormalities with CIS and therefore likely represents a precursor lesion. It is most often diagnosed in the context of known urothelial neoplasia. One study that applied the 1998 WHO/ISUP criteria indicated a 15% risk of developing cancer with a mean follow up of 4.9 years [7].

### Question No 6. *Carcinoma in situ of the urothelium. Which of the followings is wrong?*

1. The type of atypia often seen in invasive urothelial carcinoma

2. It is histologically characterized by unequivocal severe cytological atypia

3. A common feature of CIS is the lack of intercellular cohesion resulting in extensive denudation

4. Human polyoma virus infection might result in the development of CIS

#### Explanatory notes

**Figure 6:**
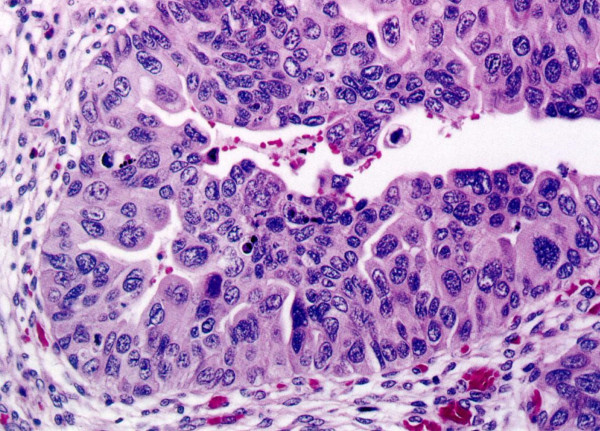
**Carcinoma in situ of the urothelium**.

Histologically CIS is characterized by architectural disorder and nuclear pleomorphism (Figure 6). The cytologically atypical cells need not involve the full thickness of the epithelium and at the minimum single malignant cells growing in a pagetoid fashion are sufficient for the diagnosis of CIS. Individual cells tend to show marked cytological atypia but increased N:C ratio is not a prerequisite (not present in the large cell type of CIS). In some cases only a few isolated cells are present clinging to the basement membrane (denuding CIS).

The classification recognizes the need to expand the category of CIS to include lesions that had been graded in the severe dysplasia category in previous systems. This change reflects current practice in major institutions treating bladder cancer. There is recognition that this is the most reproducible diagnostic category. CIS is accepted as a direct precursor of invasive carcinoma. The development of invasion is seen in the follow-up in 20 to 30% of the cases [2,5,8].

### Question No 7. Papillary urothelial neoplasm of low malignant potential. Which of the followings is correct?

1. Papillae with minimal architectural abnormalities and nuclear atypia, with more cell layers than papilloma

2. Papillary urothelial neoplasm of low malignant potential is associated with invasion or metastasis

3. Follow-up of the patient is not needed

4. Patients are not at an increased risk of developing recurrent or new papillary lesions usually of similar histology

#### Explanatory notes

**Figure 7:**
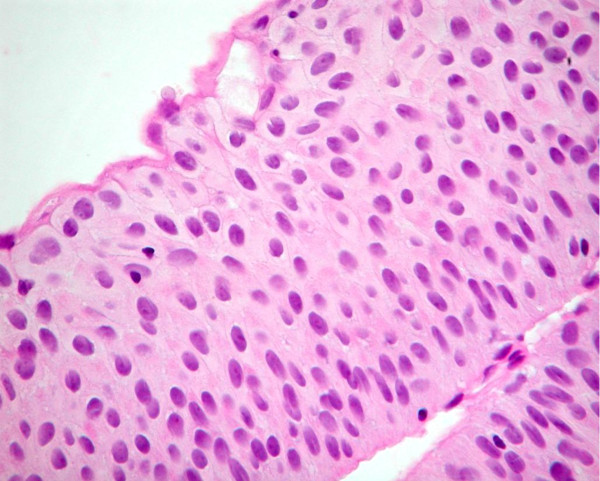
**Papillary urothelial neoplasm of low malignant potential**.

Morphologically PUNLMP largely, though not completely, corresponds to grade 1 papillary carcinoma in the old WHO system (Figure 7). The tumour consists of delicate papillae with little or no fusion. The covering urothelium shows minimal if any architectural irregularity. Nuclei are roughly normal in size, lack significant nuclear hyperchromasia or pleomorphism. The chromatin is fine and nucleoli are inconspicuous. Mitoses are infrequent and basally located. These tumors have a significantly lower rate of recurrence than either low- or high-grade papillary carcinomas and a very low rate of stage progression [5,9]. In a review of published studies, Lopez-Beltran [5,8] found the mean tumour recurrence rate to be 36% and stage progression rate to be 3.7%.

### Question No 8. *Low-grade papillary urothelial carcinoma. Which of the followings is correct?*

1. This lesion is usually associated with invasion or metastases at the time of presentation

1. Mitotic figures, including atypical forms, are frequently seen at all levels

2. Low grade papillary urothelial carcinomas have a high risk of progression, with figures varying from 15% to 40%, and of association with invasive disease at the time of diagnosis

3. The urothelium lining the papillae is similar to flat urothelial dysplasia

#### Explanatory notes

**Figure 8:**
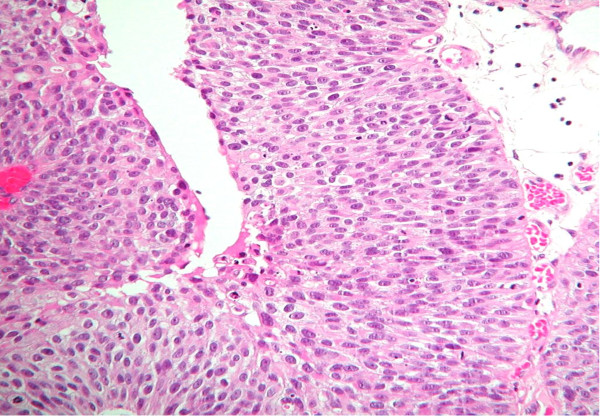
**Low-grade papillary urothelial carcinoma**.

This category contains the intermediate group of lesions. In the 1973 WHO system this would include the lower 1/2 of grade 2 papillary carcinoma. Histologically the papillae are largely delicate and separate but some fusion may be seen. At low magnification there is a generally ordered appearance to the cells within the epithelium (Figure 8). The nuclei tend to be uniformly enlarged and retain the elongated to oval shape of normal urothelial cells. The chromatin remains fine with small and generally inconspicuous nucleoli. Mitoses may be present but are few and remain basally located. The urothelium lining the papillae is similar to flat dysplasia. These tumors have a significantly higher recurrence rate than for PUNLMP and similar to high-grade papillary carcinomas. They also have a significantly higher rate of stage progression than PUNLMP but significantly lower than for high-grade papillary carcinoma [2,5,9]. A review of the literature revealed a mean recurrence rate of 50% and mean stage progression rate of 10% [5,8].

### Question No 9. *High-grade papillary urothelial carcinoma Which of the followings is correct?*

1. Mitotic figures are infrequent and usually seen in the lower half of the urothelium

2. Tumour recurrence, stage progression and tumour-related mortality occur in approximately 35%, 4% and 2% of patients, respectively

3. Tumour recurrence, stage progression and tumour-related mortality are approximately 50%, 10% and 5%, respectively

4. High grade papillary urothelial carcinomas have a high risk of progression, with figures varying from 15% to 40%, and of association with invasive disease at the time of diagnosis

#### Explanatory notes

**Figure 9:**
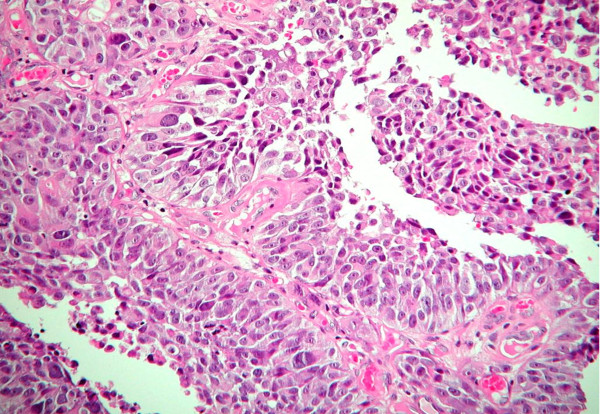
**High-grade papillary urothelial carcinoma**.

Tumors that in many cases would have been included in the 1973 WHO grade 2 category (upper 1/2) have a significant frequency of invasion and biologically have more in common with the grade 3 tumors. Histologically, the papillae are frequently fused forming apparent solid masses. The overall impression is one of disordered growth. The epithelium is of variable thickness and is similar to flat CIS (Figure 9). Individual cells are haphazardly arranged within the epithelium and have a generally discohesive nature. Nuclei are hyperchromatic and pleomorphic. The chromatin is dense, irregularly distributed and often clumped. Nucleoli may be single or multiple and are often prominent. Mitoses are generally frequent and may be seen at any level of the epithelium. These tumours not only have a risk of invasion but have a significant risk of recurrence and progression. For this reason the consensus was that these were better included in a high-grade category with the traditional WHO grade 3 neoplasms. The overall progression rate (to invasive carcinoma) ranges from 15% to 40%. These tumours, when noninvasive (pTa) likely all require additional intravesical therapy. Heterogeneity of grade is recognized in papillary lesions [2,8,10] and the consensus was that tumours should be graded on their worst part although this needs further study.

### Question No 10. *Inverted urothelial papilloma. Which of the following statements is wrong?*

1. Endophytic lesion which shares several features with exophytic urothelial papilloma

2. Rarely, hybrid cases exist with portions of the lesion resembling exophytic papilloma and others inverted urothelial papilloma

3. Urothelial carcinoma may arise within inverted urothelial papilloma

4. Urothelial nests, clusters, or single cells invading the lamina propria

#### Explanatory notes

**Figure 10:**
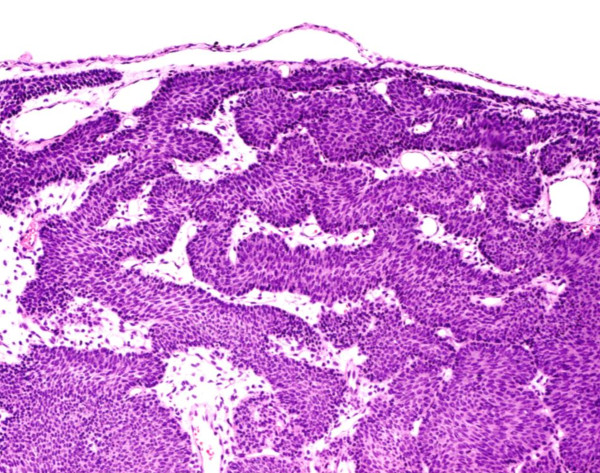
**Inverted urothelial papilloma**.

Inverted papilloma is a distinct clinical pathologic entity typically arising in the trigone region in a younger patient population than papillary neoplasms. Grossly inverted papilloma typically shows an exophytic polypoid growth pattern. Histologically it consists of anastomosing trabeculae of urothelium covered by a normal or attenuated urothelium (Figure 10). There is no significant nuclear pleomorphism and few mitoses can be seen. Squamous or glandular differentiation may be present. In TUR material the fragmentation of the lesion may result in apparent true papillary structures making diagnosis difficult. Distinction from papillary carcinoma with an inverted growth pattern can be problematic (See below). Cases of synchronous inverted papilloma and papillary carcinoma are well described. It is associated with a low risk of recurrence (< 5%) [11]. Recent genetic data supports the idea that inverted papilloma is not related to papillary urothelial neoplasms [12].

### Question No 11. *Urothelial carcinoma with endophytic growth patterns is*

1. A variant of invasive urothelial carcinoma

2. A non-invasive urothelial carcinoma exhibiting a prominent endophytic growth pattern resulting in considerable difficulty in assessing invasion

3. Any invasive urothelial carcinoma

4. Does not occur in the bladder

#### Explanatory notes

**Figure 11:**
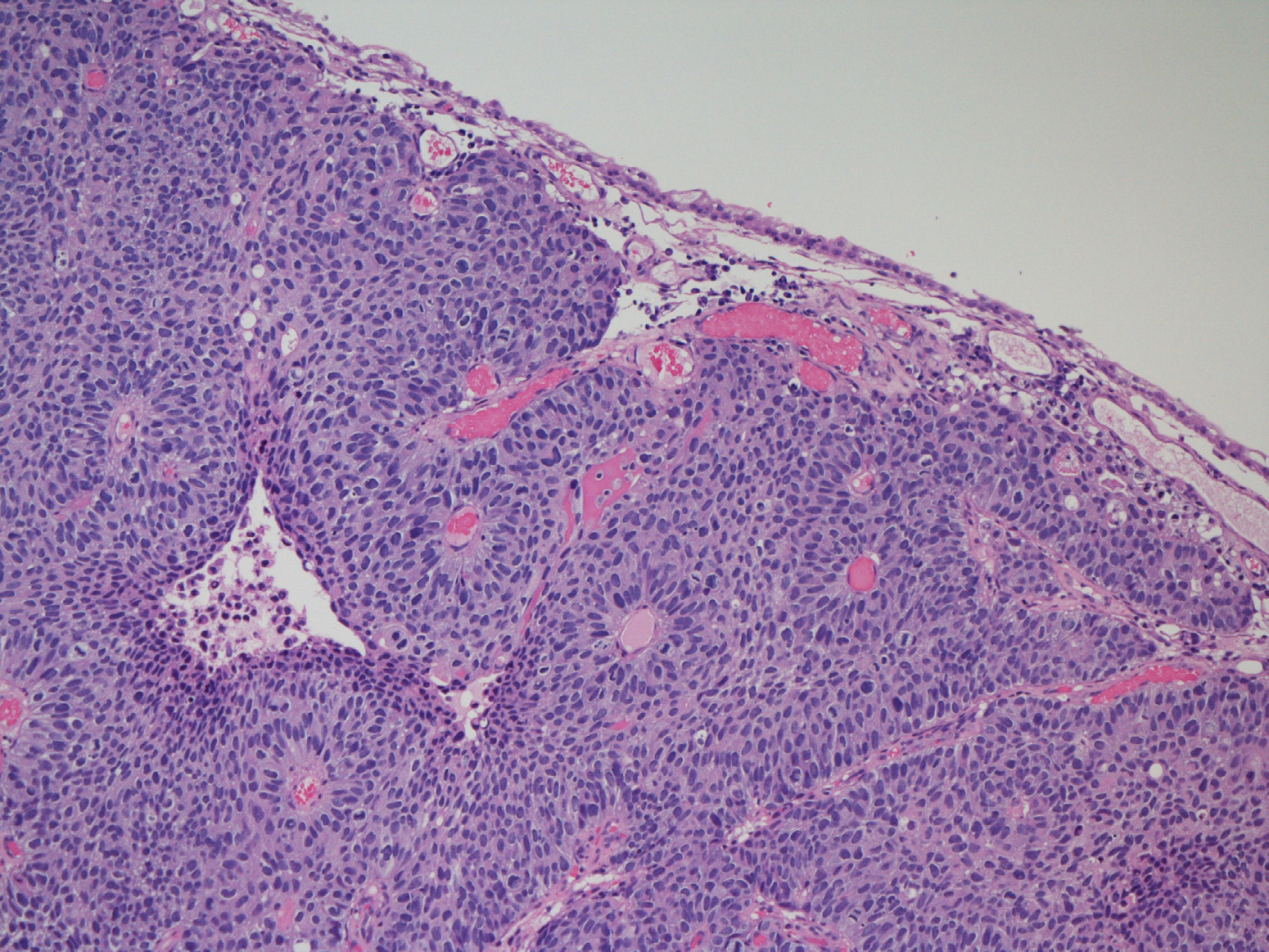
**Urothelial carcinoma with endophytic growth pattern**.

Some papillary urothelial carcinomas exhibit a prominent endophytic growth pattern resulting in considerable difficulty in assessing invasion [13]. Endophytic growth is evident either as inter-anastomosing cords and columns of urothelium, often with a striking resemblance to inverted papilloma (inverted papilloma-like pattern), or as broad, pushing bulbous invaginations into the lamina propria (broad-front pattern) (Figure 11). Distinction from inverted papilloma requires attention to architectural and cytological features. A diagnosis of invasion requires the unquestionable presence within the lamina propria of irregularly shaped nests or single cells that may have evoked a desmoplastic or inflammatory response. A stromal response may be absent. In such instances, irregularity of the contours of the invasive nests, architectural complexity, and recognition of single-cell invasion are helpful. Occasionally, the cells in the invading nests appear morphologically different from the cells at the base of the tumour, and they may appear as smaller aggregates present within empty spaces. These spaces may mimic vascular invasion closely, but they are believed to be retraction artifacts.

### Question No 12. *Urothelial carcinoma with lamina propria invasion. Which of the following statements is wrong?*

1. Urothelial nests, clusters, or single cells invading the lamina propria.

2. Often associated with a desmoplastic or inflammatory stromal response

3. Nests of invasive tumour within the lamina propria may exhibit prominent retraction artifact which is frequently overdiagnosed as vascular invasion.

4. Urothelial nests invading adipose tissue is always pT3 stage

#### Explanatory notes

**Figure 12:**
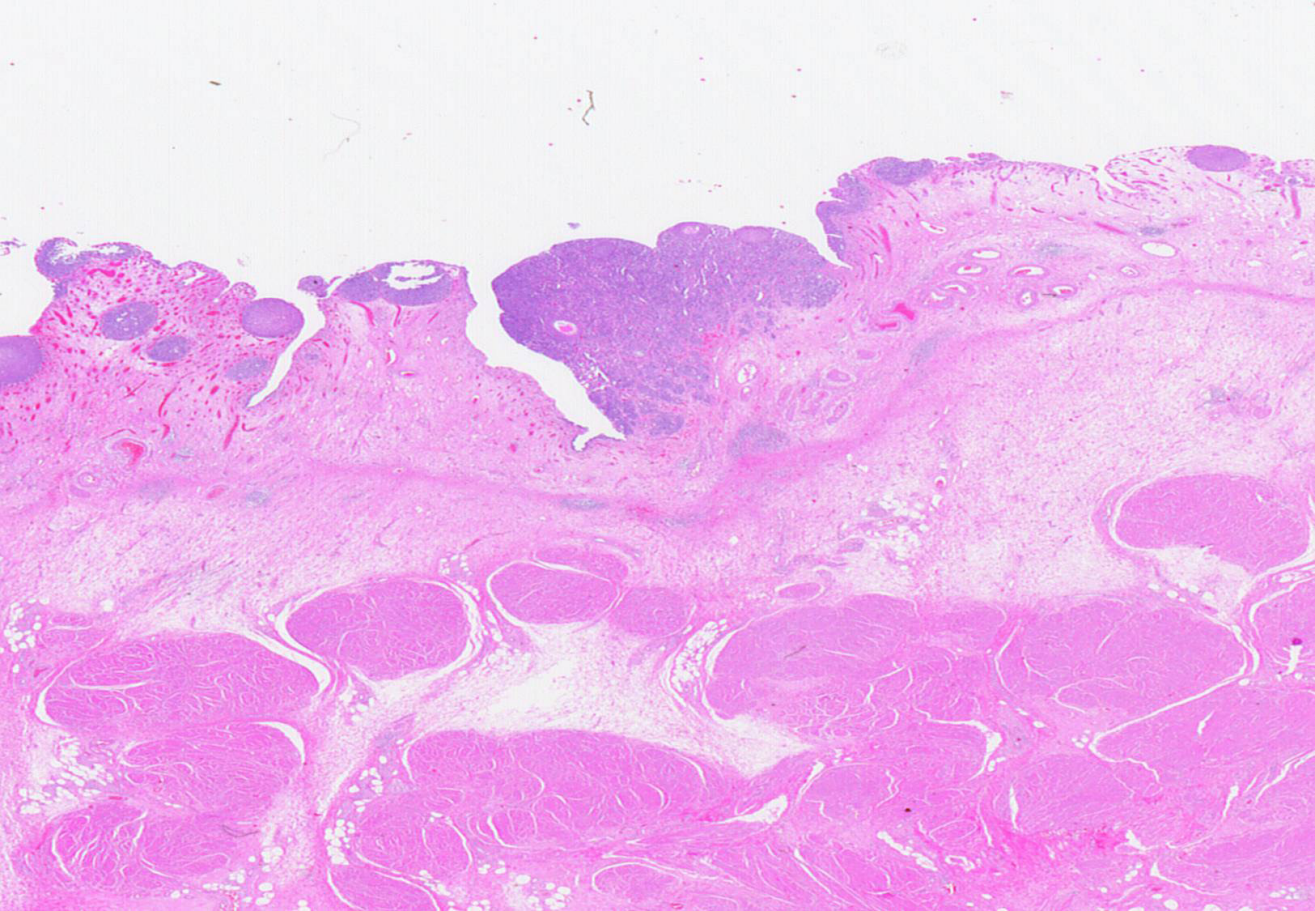
**Urothelial carcinoma with lamina propria invasion**.

The invasive front of the tumour may be seen as single cells or nests or finger-like extensions (Figure 12) [9]. The infiltrating component often shows higher degree of nuclear pleomorphism and has abundant eosinophilic cytoplasm. Stroma may show a desmoplastic or heavy inflammatory response. Retraction artifacts, mimicking vascular-lymphatic invasion, are particularly frequent in tumours superficially invading into the lamina propria and should not be overdiagnosed as vascular invasion. Vascular invasion in cases with lamina propria invasion is uncommon and should be diagnosed only in unequivocal cases or after immunohistochemistry [14]. In the absence of stromal response the diagnosis of invasion rely on the characteristics of the infiltrating epithelium.

Recently-emphasized pitfalls in the diagnosis of lamina propria invasive urothelial carcinoma include tangential sectioning, thermal artifact, obscuring inflammation, CIS involving von Brunn nests, deceptively bland tumours such as microcystic and nested urothelial carcinoma variants, invasion into indeterminate type of muscle, and invasion into adipose tissue within lamina propria [14,15]. Determination of the type of muscle (muscularis mucosae vs. muscularis propria) invaded by carcinoma can be particularly difficult due to different problems including: small sample size, tissue distortion, cautery artifact, poor orientation, fibrosis and inflammation elicited by destructive growth of invasive tumour, even hypertrophy of the normally thin and discontinuous muscularis mucosae. In those cases the designation of "muscle type indeterminate" is a viable description [16]. Only in the last few years has the presence of adipose tissue in all layers of the bladder including lamina propria and muscularis propria been well-documented [17]. Thus the presence of carcinoma in fat does not necessarily indicate extra-vesical extension. Invasive tumour should be graded as low-or high-grade analogous to the scheme used for grading non-invasive lesions.

### Question No 13. *Urothelial carcinoma with muscularis propria invasion. Which of the statements is wrong*

1. Tumour cells infiltrate thick muscle bundles

2. Urothelial nests, clusters, or single cells within the subepithelial connective tissue

3. Is much more aggressive than low-grade carcinoma

4. In a TUR specimen there should be no attempt to substage the depth of muscularis propria invasion

#### Explanatory notes

**Figure 13:**
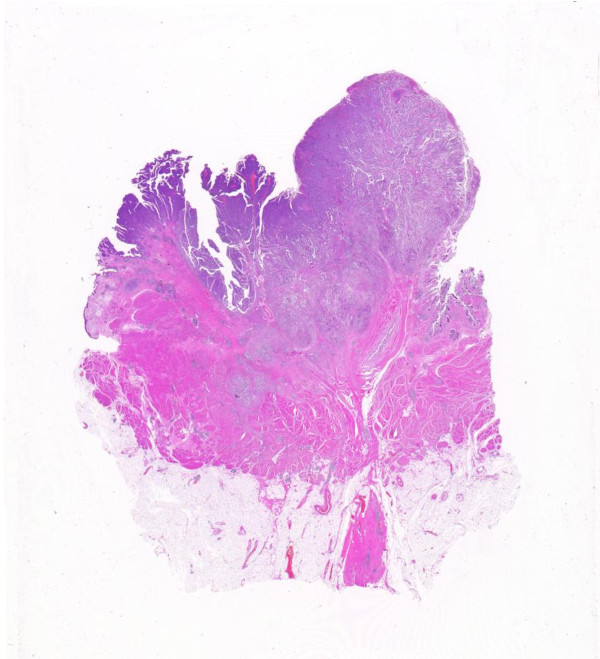
**Urothelial carcinoma with muscolaris propria invasion**.

(See also comments related to MCQ 12) The level of invasion of the lamina propria is related to patient outcome, with a worse prognosis for tumours that invade beyond the muscularis mucosae (pT1b) [14] or deeply into the subepithelial connective tissue, as quantitated using an ocular micrometer. The WHO 2004 group recommended that some estimate of extent of lamina propria invasion (for example: pT1a - above or into muscularis mucosae vs. pT1b - tumours below) (Figure 13) [2] be provided but this is currently not a formal part of the 2002 TNM system, and it is not universally reported, since there is no established method that is consistently applicable and reproducible. In fact it is often difficult to identify the depth of lamina propria invasion due to the lack of orientation in the transurethral resection (TUR) chips or because of the absence of muscularis mucosae and thick-walled vessels. Nevertheless, pathologists are encouraged to provide some assessment as to the extent of lamina propria invasion. If the tumour invades muscularis mucosae, it should be mentioned in the report unambiguously so that the urologist does not confuse muscularis mucosae with muscularis propria. The presence or absence of muscularis propria in the specimen should always be mentioned, even in cases of noninvasive disease, with the purpose of giving feedback to the urologist as to the depth of the biopsy [14].

### Question No 14. *Urothelial carcinoma with muscularis propria invasion. Which of the statements are correct*

1. Substaging of pT2 (pT2a, invasion of "superficial" muscle or inner half, vs. pT2b, invasion of deep muscle or outer half) can only been done on radical cystectomy specimens

2. Muscularis mucosae invasion is synonymous to muscularis propria invasion

3. The presence or absence of muscularis propria in biopsy and transurethral resection of bladder tumour samples, and presence or absence of carcinoma in identified muscularis propria, should not be specified

4. Clinicians should accept a diagnosis of "Urothelial carcinoma invading muscle" without demanding further clarification from the pathologist

#### Explanatory notes

Invasion by bladder carcinoma into muscularis propria (muscle wall, detrusor) is an ominous finding where the patient becomes a candidate for radical cystectomy or radiation therapy with or without adjuvant chemotherapy. Special studies, such as a Masson stain or immunohistochemistry with antibodies to actin, help identify all smooth muscle tissue. The highlighting of numerous muscle fibres distributed throughout an extensive tumour may lead to a diagnosis of muscularis propria invasion [14]. Situations where there is uncertainty as to the presence muscularis propria invasion should be conveyed to the urologist. Clinicians should not accept a diagnosis of "TCC invading muscle" without demanding further clarification (if possible) from the pathologist.

The presence or absence of muscularis propria in biopsy and transurethral resection of bladder tumour (TURBT) samples, and presence or absence of carcinoma in identified muscularis propria, should be specified.

Substaging of pT2 (pT2a - invasion of "superficial" muscle = inner half vs. pT2b - invasion of "deep" muscle = outer half) and distinction of pT2 vs. pT3 can only been done on radical cystectomy specimens, and not TURBT samples (Figure 1I, insert) [14]. Even in cystectomy specimens it can be a challenge at times to determine extra-vesical (pT3) spread since the boundary between muscularis propria and its fat is not well-demarcated from peri-vesical fat. Moreover, this boundary can be distorted, obscured, or obliterated by fibrosis and inflammation associated with infiltrating tumour.

### Question No 15. *Which of the following statements is wrong*

1. The WHO 2004 classification is the most current version in bladder tumour classification.

2. The WHO 1973 classification is still considered by many urologists and oncologists as the international standard in patient's management.

3. The TNM system should be preferred to the WHO 2004 classification

4. Lesions called WHO 1973 grade 3 are by definition high-grade carcinoma in the WHO 2004 system

#### Explanatory notes

A major misconception is that there is a one to one translation between the 1973 and 2004 WHO classification systems. Only at the extremes of grades in the 1973 WHO classification, does this correlation hold true. Lesions called papilloma in the WHO classification system would also be called papilloma in the 2004 WHO system. At the other end of the grading extreme, lesions called WHO grade 3 are by definition high-grade carcinoma in the 2004 WHO system. However, for WHO grades 1 and 2, there is no direct translation to the WHO/ISUP system [5].

## Supplementary Material

Additional file 1 **APPENDIX 1**. 2004 WHO classification of the urothelial neoplasms Click here for file

Additional file 2 **Appendix 2**. Recording form. Click here for file
