# Supplementary material for: How much do you know about benign, preneoplastic, non-invasive and invasive neoplastic lesions of the urinary bladder classified according to the 2004 WHO scheme?
Source: Diagn Pathol. 2011 Apr 7;6:31. doi: 10.1186/1746-1596-6-31 (PMC3107770; doi:10.1186/1746-1596-6-31)
Supplement: Additional file 1 — APPENDIX 1. 2004 WHO classification of the urothelial neoplasms [file 1746-1596-6-31-S1.doc]

**APPENDIX 1. 2004 WHO classification of the urothelial neoplasms**

# NORMAL

# Normal*

# HYPERPLASIA

#

# Flat Hyperplasia

# Papillary Hyperplasia

# FLAT LESIONS WITH AYTPIA

# Reactive (Inflammatory) Atypia

# Atypia of Unknown Significance

# Dysplasia (Low-grade Intraurothelial Neoplasia)

# Carcinoma In Situ (High-grade Intraurothelial Neoplasia)**

#

# PAPILLARY NEOPLASMS

# Papilloma

# Inverted Papilloma

# Papillary Neoplasm of Low Malignant Potential

# Papillary Carcinoma, Low grade

# Papillary Carcinoma, High grade

#

# INVASIVE NEOPLASMS

# Lamina Propria Invasion

# Muscularis Propria (Detrusor Muscle) Invasion

# *May include cases formerly diagnosed as “mild dysplasia”

# ** Includes cases with “severe dysplasia”
