# Supplementary material for: How much do you know about benign, preneoplastic, non-invasive and invasive neoplastic lesions of the urinary bladder classified according to the 2004 WHO scheme?
Source: Diagn Pathol. 2011 Apr 7;6:31. doi: 10.1186/1746-1596-6-31 (PMC3107770; doi:10.1186/1746-1596-6-31)
Supplement: Additional file 2 — Appendix 2. Recording form. [file 1746-1596-6-31-S2.doc]

**Appendix 2. Recording form**

**Question No 1. *Normal urothelium. Which of the followings items is wrong?***

Answer:

Comments:

**Question No 2. *Flat urothelial hyperplasia. Which of the following items is correct?***

Answer:

Comments:

**Question No 3. *Reactive atypia. Which of the followings is correct?***

Answer:

Comments:

**Question No 4. *Papillary urothelial hyperplasia (Pseudopapillary hyperplasia). Which of the followings is correct?***

Answer:

Comments:

**Question No 5. *Urothelial dysplasia Which of the followings is correct?***

Answer:

Comments:

**Question No 6. *Carcinoma in situ of the urothelium. Which of the followings is wrong?***

Answer:

Comments:

**Question No 7**. ***Papillary urothelial neoplasm of low malignant potential. Which of the followings is correct?***

Answer:

Comments:

**Question No 8. *Low-grade papillary urothelial carcinoma. Which of the followings is correct?***

Answer:

Comments:

**Question No 9. *High-grade papillary urothelial carcinoma Which of the followings is correct?***

Answer:

Comments:

**Question No 10**. ***Inverted urothelial papilloma. Which of the following statements is wrong?***

Answer:

Comments:

**Question No 11. *Urothelial carcinoma with endophytic growth patterns is:***

Answer:

Comments:

## Question No 12. *Urothelial carcinoma with lamina propria invasion. Which of the following statements is wrong?*

Answer:

Comments:

## Question No 13. *Urothelial carcinoma with muscularis propria invasion. Which of the statements is wrong:*

Answer:

Comments:

## Question No 14. *Urothelial carcinoma with muscularis propria invasion. Which of the statements are correct:*

Answer:

Comments:

## Question No 15. *Which of the following statements is wrong:*

Answer:

Comments:
